# Overexpression of Lifeact-GFP Disrupts F-Actin Organization in Cardiomyocytes and Impairs Cardiac Function

**DOI:** 10.3389/fcell.2021.746818

**Published:** 2021-10-26

**Authors:** Rui Xu, Shaojun Du

**Affiliations:** Department of Biochemistry and Molecular Biology, Institute of Marine and Environmental Technology, University of Maryland School of Medicine, Baltimore, MD, United States

**Keywords:** F-actin filament, Lifeact-GFP, sarcomere, cardiomyocyte, Smyd1

## Abstract

Lifeact-GFP is a frequently used molecular probe to study F-actin structure and dynamic assembly in living cells. In this study, we generated transgenic zebrafish models expressing Lifeact-GFP specifically in cardiac muscles to investigate the effect of Lifeact-GFP on heart development and its application to study cardiomyopathy. The data showed that transgenic zebrafish with low to moderate levels of Lifeact-GFP expression could be used as a good model to study contractile dynamics of actin filaments in cardiac muscles *in vivo*. Using this model, we demonstrated that loss of Smyd1b, a lysine methyltransferase, disrupted F-actin filament organization in cardiomyocytes of zebrafish embryos. Our studies, however, also demonstrated that strong Lifeact-GFP expression in cardiomyocytes was detrimental to actin filament organization in cardiomyocytes that led to pericardial edema and early embryonic lethality of zebrafish embryos. Collectively, these data suggest that although Lifeact-GFP is a good probe for visualizing F-actin dynamics, transgenic models need to be carefully evaluated to avoid artifacts induced by Lifeact-GFP overexpression.

## Introduction

Actin is one of the most abundant proteins in eukaryotic cells and exists as either a free monomer called G-actin (globular) or a linear polymer microfilament called F-actin (filamentous). The F-actin is an important component of the cytoskeleton in eukaryotic cells and thin filaments in myofibrils of muscle cells. F-actin participates in many important cellular processes, including cell division, intracellular cargo transport, cell migration, cell morphogenesis, and muscle contraction (Pollard and Cooper, 2009; Dominguez and Holmes, 2011). A large number of illnesses and diseases are caused by genetic mutations in actin and its associated proteins. In muscle cells, actin thin filaments are an essential part of the sarcomere structure, the basic unit of muscle contraction. Actin thin filaments work together with myosin thick filaments to drive muscle contraction in heart and skeletal muscles. Defective thin filament assembly from actin mutations is associated with actin-accumulation myopathy, nemaline myopathy, and cardiomyopathy (Nowak et al., 1999; Costa et al., 2004; Despond and Dawson, 2018; Frustaci et al., 2018).

Visualization of actin filament structures and dynamic assembly in living cells is critical for the study of various cellular processes of cell division, migration, polarization, and muscle contraction. Several different techniques and approaches are currently applied to analyze and visualize cellular actin structures and their dynamic assembly and disassembly in various cell and model systems (Belin et al., 2014; Lemieux et al., 2014; Spracklen et al., 2014; Du et al., 2015). Phalloidin is the standard F-actin marker commonly used to label F-actin in fixed samples and tissues. However, phalloidin has a toxic side effect to living cells (Wehland et al., 1977; Cooper, 1987), thus limiting its application in live cell imaging. Live-cell analysis of actin filaments and their dynamic assembly largely relies on genetically modified GFP derivatives that tag actin directly or actin-binding domains (Melak et al., 2017). Tagging actin directly using GFP is a simple and popular technique, which has been successfully used for studying actin dynamics and turnover within a given cellular structure using the fluorescence recovery after photobleaching (FRAP) approach (Koestler et al., 2009; Clark et al., 2013). However, the relatively large size of GFP-tagged actin can give rise to problems in terms of the incorporation of Actin-GFP monomers into filaments. In addition, the Actin-GFP chimeras can interfere with the normal functionality of actin cytoskeleton resulting in experimental artifacts (Aizawa et al., 1997; Nagasaki et al., 2017).

To avoid the problem of steric clashes with actin from using large and bulky Actin-GFP chimeras, small fluorophore labeled peptides, such as Lifeact-GFP, were developed. Lifeact-GFP is the most popular and widely used actin probe for visualizing F-Actin structures in various cell types and model organisms. Lifeact-GFP is generated by fusing a short 17-amino-acid peptide (Lifeact) from yeast Abp140 with GFP (Riedl et al., 2008; Fraccaroli et al., 2012; Reischauer et al., 2014; Higuchi-Sanabria et al., 2018). The specific and selective binding of Lifeact with F-actin permits the study of intracellular actin in the native environment without significantly interfering with actin dynamics *in vitro* or *in vivo*. Thus, Lifeact-GFP surpasses other reagents like fluorescent phalloidin, actin-GFP, and anti-actin antibodies. It enables live imaging of the actin cytoskeleton and therefore the study of many fundamental biological processes *in vivo*.

Numerous studies have been published describing the application of Lifeact in studying cell motility (Duleh and Welch, 2012; Fiolka et al., 2012; Rullo et al., 2012; Grikscheit et al., 2015; Suarez et al., 2015). Cells expressing Lifeact-GFP showed no defects of actin dynamics, cell physiology, or tissue organization, suggesting that Lifeact-GFP expression does not interfere with cellular processes. Transgenic mice expressing Lifeact-GFP that were viable, phenotypically normal, and fertile have been successfully generated (Riedl et al., 2010), thus validating Lifeact-EGFP as a reporter that doesn’t interfere with physiological processes. Additionally, Lifeact-GFP has been successfully employed to generate other transgenic models including *Arabidopsis* (Vidali et al., 2009; Van Der Honing et al., 2011), Drosophila (Zanet et al., 2012; Huelsmann et al., 2013), and zebrafish (Phng et al., 2013; Reischauer et al., 2014; Xu et al., 2014; Fukuda et al., 2017). These Lifeact-EGFP transgenic models provide new opportunities to study actin remodeling in all cells, within native tissues.

Although it was believed that Lifeact-GFP does not interfere with actin dynamics *in vitro* or *in vivo*, several recent reports have raised concerns on Lifeact-associated artifacts at the cellular and whole organismal levels. It has been reported that overexpression of Lifeact caused infertility and severe actin defects and multiple nuclei in follicle cells in Drosophila (Spracklen et al., 2014). Similarly, strong Lifeact expression disturbed actin assembly in fission yeast (Courtemanche et al., 2016). Recent studies demonstrated that Lifeact altered cell cytoskeleton and morphology in mammalian cells (Flores et al., 2019). Most of the Lifeact-GFP induced artifacts were observed in actin cytoskeleton structures in non-muscle cells. It is not clear whether Lifeact-GFP can impede actin thin filaments in sarcomeres of skeletal and cardiac muscle cells.

In addition to Lifeact, actin binding domains of F-tractin, UtrCH and Fimbrin have been successfully used in constructing actin probes for live imaging (Melak et al., 2017). F-tractin contains a 43-amino-acid long peptide from the rat actin-binding inositol 1,4,5-trisphosphate 3-kinase A (Schell et al., 2001; Belin et al., 2014), whereas UtrCH comprises the first 261 amino acids of human utrophin, an actin-binding protein (Winder et al., 1995). Fimbrin on the other hand is an actin cross-linking protein, important in filopodia formation (Bretscher, 1981; Matsudaira, 1994). The actin binding domains in these proteins fused with GFP have been successfully used for imaging actin filaments in a wide range of organisms and cell types (Sheahan et al., 2004; Wang et al., 2004; Burkel et al., 2007; Holubcová et al., 2013).

SiR-actin and Actin-Chromobody are two recently introduced actin markers. SiR-actin is a cell-permeable chemically synthesized probe, a structural analog of F-actin-binding toxin from a marine sponge (Bubb et al., 1994; Lukinavičius et al., 2014; D’Este et al., 2015). Advantages of SiR-actin are its ease of use in live imaging as it avoids the time-consuming steps of cell transfection and protein overexpression. However, SiR-actin can cause F-actin stabilization or induce actin polymerization, making interpretations of observed F-actin structures more difficult. In comparison, it appears that high levels of Actin-Chromobody expression does not alter actin dynamics. Actin chromobodies are fluorescent-protein-tagged actin nanobodies that have been successfully used for imaging sub-organellar actin dynamics in cultured cells and live imaging of endogenous protein dynamics in zebrafish (Panza et al., 2015; Schiavon et al., 2020).

Zebrafish embryos, with their optical transparency, offer the potential to image contractile structures in skeletal and cardiac muscles *in vivo*. Transgenic zebrafish expressing Lifeact-GFP reporter have been generated and successfully used for a high-resolution imaging analysis of actin myofibril dynamics in the beating heart of zebrafish embryos and for studying cardiomyopathy from drug exposure and genetic mutations (Reischauer et al., 2014; Fukuda et al., 2017). The potential adverse effects of Lifeact-GFP overexpression on the heart have not been investigated. In this study, we generated Tg(myl7:Lifeact-GFP) transgenic zebrafish models to investigate the effect of Lifeact-GFP expression on heart development and the use of Lifeact-GFP to study cardiomyopathy. We showed that transgenic zebrafish with low to moderate levels of Lifeact-GFP expression could serve as a useful model to study contractile dynamics of actin filaments in cardiac muscles *in vivo*. Using this model, we found that loss of *smyd1b* function resulted in severe actin filament disassembly in cardiac myocytes leading to pericardial edema in zebrafish embryos. Moreover, our studies also demonstrated that strong cardiac expression of Lifeact-GFP was detrimental to actin filament organization in cardiomyocytes of zebrafish embryos resulting in pericardial edema and early embryonic lethality. Collectively, these data demonstrate that the use of Lifeact requires a more elaborate evaluation and optimization to avoid Lifeact-GFP induced artifacts.

## Materials and Methods

### Ethics Statement

This study was carried out in accordance with the recommendations in the Guide for the Care and Use of Laboratory Animals of the National Institutes of Health. To ease pain and facilitate animal handling, fish embryos over one day old were anaesthetized in 0.6 mM Tricaine (pH 7.0) before fixation in 4% paraformaldehyde (PFA) for whole mount observation and immunostaining.

### Zebrafish Lines and Maintenance

All adult zebrafish were kept at the zebrafish facility at the Institute of Marine and Environmental Technology, University of Maryland. Zebrafish larvae and adult were maintained at 28.5°C in a recirculating aquatic system at a photoperiod of 14-h light and 10 h dark cycle. The *smyd1b*^*sa15678*^ mutant was obtained from ZIRC (Busch-Nentwich et al., 2013). The *Tg(myl7:Lifeact-GFP)* and *Tg(acta1b:Lifeact-GFP*) constructs used to generate the transgenic lines and transient expression assay were gifts from Dr. Didier Stainier (Reischauer et al., 2014). The *pTol2-*α*-actin-EGFP* plasmid was constructed in Tol2 vector using *α-actin* promoter to drive EGFP expression (Li et al., 2020).

### Transgenic Fish Generation and Transient Gene Expression by Microinjection

DNA constructs of *Tg(myl7:Lifeact-GFP), Tg(acta1b:Lifeact-GFP*) and *pTol2-*α*-actin-EGFP* were dissolved in sterile water to a final concentration of 50 or 100 ng/μl. For transient expression analysis, approximately 1–2 nl of DNA construct (50–100 pg) was injected into each embryo at one- or two-cell stage. For the generation of transgenic zebrafish lines, the DNA constructs were mixed with Tol2 transposase mRNA (50 ng/μl). Approximately 1–2 nl of mixed DNA construct/Tol2 transposase mRNA was injected into each embryo at one- or two-cell stage. Transgenic zebrafish founders (P1) were screened by examining GFP expression in their F1 embryos at 24 hpf under a fluorescence microscope (Axioplan 2, Zeiss).

### Antibody and Phalloidin Staining

Immunostaining of zebrafish embryonic hearts was carried out at 3 and 14 days-post-fertilization (dpf) as described previously (Yang and Xu, 2012). In brief, the embryos were fixed with 4% formaldehyde. The hearts were dissected with forceps, and then stained with α-actinin (clone EA-53, A7811; MilliporeSigma) primary antibody, and visualized using goat anti-mouse Alexa Fluor-555 conjugated secondary antibody. To increase penetration, hearts from 2-weeks old larvae was treated with 0.1% CHAPS overnight before adding primary antibodies. Phalloidin staining was carried out on the dissected hearts by incubation with 20 ng/ml phalloidin-TRITC (P1951; MilliporeSigma) for 1 h at room temperature in the dark. The images were photographed using a confocal microscope (SP8; Leica Microsystems, Buffalo Grove, IL, United States).

### Inverse PCR

Inverse PCR was performed as described (Kawakami et al., 2004; Kawakami, 2007) with some modifications. Genomic DNA was isolated from 30 transgenic embryos at 5 dpf using the DNeasy Blood & Tissue Kits (Qiagen). A 1 μg of genomic DNA was digested with Alul or *Hae*III, and then circularized by ligation. The ligated circular DNA was purified and used for two rounds of nested PCR to isolate the sequence at the 5′ junction of the transgene. The first round of PCR was carried out using Tol2-5′/f1 (5′-AAGTACTTTTTACTCCTTACAA-3′) and Tol2-5′/r1 5′-TGATTTTTAATTGTACTCAAGT-3′) primers. The second round was performed using Tol2-5′/f2 (5′- TTACAGTCAAAAAGTACTTA-3′) and Tol2-5′/r2 (5′-CAAGTAAAGTAAAAATCCC-3′) primers. The PCR products were purified and cloned into pGEM-T easy for sequencing.

### Morpholino Oligonucleotide (MO) Knockdown

The standard control-MO and the Lifeact-GFP-MO (GATCAAATCTGCGACACCCATCCCC) targeted to the ATG start site of Lifeact were purchased from Gene Tools (Corvallis, OR, United States). The morpholino antisense oligos were dissolved in 1 × Danieau buffer (Nasevicius and Ekker, 2000) to a final concentration of 0.5 mM. Zebrafish embryos at the 1 or 2 cell stage were injected with approximately 2 nl (10 ng) of the MOs.

### RNA Isolation and qPCR Analysis

Total RNA was isolated from transgenic larvae at 5 days post-fertilization (dpf) using Trizol (Invitrogen, United States). Three biological replicates were carried out for each line. The RNA samples were treated with RNase-free DNase (Promega, United States). For each sample, 0.5 μg of RNA was used for reverse transcription using Maxima First Strand cDNA Synthesis Kit (Thermo Fisher, United States). qPCR was performed on the Applied Biosystems QuantStudio 3 (Thermo Fisher, United States) using the PowerUp^*TM*^ SYBR^*TM*^ Green Master Mix (Thermo Fisher, United States). The GFP and elongation factor 1α (EF1α) specific primers are: GFP-qpcr-F (5-CAAGCAGAAGAACGGCATCAAGGTG-3), GFP-qpcr-R (5-G GACTGGGTGCTCAGGTAGTGGTTG-3), EF1α-P3 (5′ – CTT CAACGCTCAGGTCATCAT – 3′) and EF1α-P4 (5′ – ACAGC AAAGCGACCAAGAGGA – 3′). The PCR was performed with the following parameters including 2 min initial activation at 50°C and followed by 40 cycles of PCR reaction. Each cycle includes 15 s denaturation at 95°C, 10 s annealing at 64°C, and 20 s elongation at 72°C. For each qPCR experiment, samples were run with three technical replicates. The relative amount of a given mRNA was calculated by using the formulae 2^–ΔΔCt^ with EF1α as reference.

### Image-Based GFP Intensity Measurements

GFP fluorescent images were collected under a fluorescent microscopy (Leica Microsystems) with the same parameters. For each transgenic line, three embryos were randomly chosen for imaging at 24 h post-fertilization (hpf). ImageJ was used to manually measure GFP fluorescent intensity for each heart tube.

### Statistical Analysis

Data are presented as the mean ± sd. Statistical differences were analyzed by using a student’s *t* test. *P* value < 0.05 was set as the threshold for statistical significance.

## Results

### Development of *Tg(myl7:Lifeact-GFP)* Transgenic Models to Investigate Congenital Cardiomyopathy in *smyd1b*^*sa15678*^ Mutant

**FIGURE 1:**
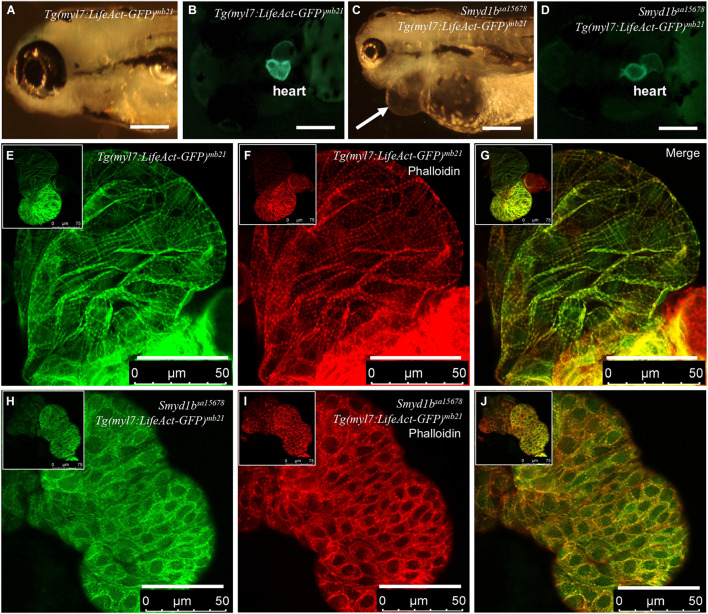
Analysis of congenital cardiomyopathy in *smyd1b*^*sa15678*^ mutant using *Tg(myl7:Lifeact-GFP)* transgenic zebrafish model. **(A–D)** Morphology and Lifeact-GFP expression in the heart region of WT *Tg(myl7:Lifeact-GFP)^*mb21*^* **(A,B)**, and *smyd1b*^*sa15678*^; *Tg(myl7:Lifeact-GFP)^*mb21*^* mutant **(C,D)** transgenic larvae at 72 hpf. Pericardial edema is indicated by arrow in *smyd1b*^*sa15678*^; *Tg(myl7:Lifeact-GFP)^*mb21*^* transgenic mutant larvae **(C)**. Scale bars: 250 μm. **(E–J)** Actin think filaments in cardiomyocytes revealed by Lifeact-GFP **(E,H)** and phalloidin staining **(F,I)** in WT *Tg(myl7:Lifeact-GFP)^*mb21*^* **(E–G)**, and *smyd1b*^*sa15678*^; *Tg(myl7:Lifeact-GFP)^*mb21*^* transgenic mutant **(H–J)** embryos at 72 hpf. **(G,J)** are merged pictures of **(E,F,H,I)**, respectively. Scale bars: 50 μm.

Previous studies demonstrated that Smyd1 lysine methyltransferase plays a vital role in heart development and myofibril assembly in cardiac muscles (Gottlieb et al., 2002; Tan et al., 2006; Just et al., 2011; Li et al., 2013; Jiao et al., 2021). Genetic mutations in human *SMYD1* are associated with congenital and hypertrophic cardiomyopathies in human (Coyan et al., 2019; Fan et al., 2019). To assess the application of Lifeact-GFP reporter in analysis of actin filament assembly in *smyd1b*^*sa15678*^ mutant with heart defects, we generated transgenic zebrafish expressing Lifeact-GFP specifically in cardiac myocytes using the *Tg(myl7:Lifeact-GFP)* transgene (Reischauer et al., 2014). Six transgenic lines were generated from eight transgenic founders (Figures 1A–D). Consistent with the previous report (Reischauer et al., 2014), Lifeact-GFP clearly revealed F-actin myofibril structures in cardiac myocytes of *Tg(myl7:Lifeact-GFP)^*mb21*^* zebrafish embryos (Figure 1E). Co-staining with phalloidin-TRITC revealed that Lifeact-GFP co-localized with Phalloidin staining at the F-actin thin filaments of cardiomyocytes (Figures 1F,G).

The *Tg(myl7:Lifeact-GFP)^*mb21*^* transgenic line that showed normal development was selected to cross with *smyd1b*^*sa15678/+*^ mutant to generate *smyd1b*^*sa15678/+*^; *Tg(myl7:Lifeact-GFP)^*mb21*^* heterozygous transgenic mutants. The *smyd1b*^*sa15678/+*^; *Tg(myl7:Lifeact-GFP)^*mb21*^* heterozygous transgenic mutants were subsequently in-crossed to generate *smyd1b*^*sa15678*^; *Tg(myl7:Lifeact-GFP)^*mb21*^* homozygous transgenic mutant embryos (Figure 1C,D). Lifeact-GFP expression was detected in cardiac muscle of wildtype (WT) and *smyd1b*^*sa15678*^ mutant transgenic embryos (Figures 1B,D). However, in contrast to WT *Tg(myl7:Lifeact-GFP)^*mb21*^* transgenic embryos (Figures 1E–G), Lifeact-GFP and phalloidin staining showed little or no F-actin myofibrils in cardiomyocytes of *smyd1b*^*sa15678*^ homozygous mutant embryos (Figures 1H–J). Lifeact-GFP and phalloidin-TRITC staining appeared as punctate in cardiomyocytes of *smyd1b*^*sa15678*^; *Tg(myl7:Lifeact-GFP)^*mb21*^* mutant transgenic fish embryos (Figures 1H–J). The homozygous transgenic mutant embryos died around 5 dpf. Together, these data indicate that Smyd1b is required for F-actin myofibril organization in cardiomyocyte, and *Tg(myl7:Lifeact-GFP)^mb21^* transgenic zebrafish are a useful model to analyze F-actin myofibril defects in cardiomyopathy.

### Lifeact-GFP Overexpression in Cardiac Muscle Cells Causes Pericardial Edema and Embryonic Lethality

**FIGURE 2:**
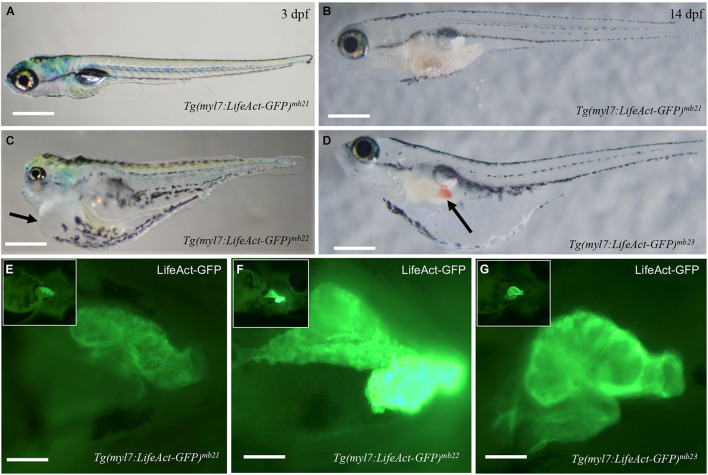
Development of pericardial edema in *Tg(myl7:Lifeact-GFP)^*mb22*^* and *Tg(myl7:Lifeact-GFP)^*mb23*^* transgenic embryos. **(A–D)** Morphology of representative transgenic embryos from *Tg(myl7:Lifeact-GFP)^*mb21*^* line with no edema **(A,B)**, and *Tg(myl7:Lifeact-GFP)^*mb22*^* **(C)**, and *Tg(myl7:Lifeact-GFP)^*mb23*^* **(D)** transgenic lines with edema phenotypes at 3 **(C)** and 14 dpf **(D)**, respectively. Pericardial edema is indicated by arrows in *Tg(myl7:Lifeact-GFP)^*mb22*^* **(C)**, and *Tg(myl7:Lifeact-GFP)^*mb23*^* **(D)** transgenic larvae. Scale bars: 500 μm. **(E–G)** Lifeact-GFP expression in the hearts of *Tg(myl7:Lifeact-GFP)^*mb21*^* **(E)**, *Tg(myl7:Lifeact-GFP)^*mb22*^* **(F)**, and *Tg(myl7:Lifeact-GFP)^*mb23*^* **(G)** transgenic embryos at 3 dpf. Scale bars: 100 μm.

*Tg(myl7:Lifeact-GFP)* transgenic embryos from six out of the eight transgenic founders showed normal development (Figures 2A,B). They were able to grow into viable fertile adults. However, two transgenic founders, *Tg(myl7:Lifeact-GFP)^*mb22*^* and *Tg(myl7:Lifeact-GFP)^*mb23*^*, gave F1 transgenic embryos with pericardial edema and died at early larval stages (Figures 2C,D). All transgenic embryos (*n* = 23) from the *Tg(myl7:Lifeact-GFP)^*mb22*^* founder showed severe edema phenotype and were 100% embryonic lethal around 6 dpf. The non-transgenic sibling from the same *Tg(myl7:Lifeact-GFP)^*mb22*^* transgenic founder were normal. In contrast, transgenic embryos from the *Tg(myl7:Lifeact-GFP)^*mb23*^* founder showed normal cardiac morphology at early stage. However, they developed an edema phenotype around 14 dpf (Figure 2D). Visible blood clots began to form in the yolk near the swimming bladder (Figure 2D). All *Tg(myl7:Lifeact-GFP)^*mb23*^* transgenic larvae died around 25–30 dpf. Together, these data suggest that expression of Lifeact-GFP might have an adverse effect on heart development.

To determine whether the pericardial edema phenotype correlated with higher levels of Lifeact-GFP expression, we compared the fluorescent intensity of Lifeact-GFP expression in *Tg(myl7:Lifeact-GFP)^*mb22*^* and *Tg(myl7:Lifeact-GFP)^*mb23*^* transgenic embryos that showed edema phenotypes (Figures 2C,D,F,G) with transgenic embryos from the *Tg(myl7:Lifeact-GFP)^*mb21*^* line that showed no edema phenotype (Figures 2A,B,E). The data revealed a clear correlation between the severity of the edema phenotype and the increased levels of Lifeact-GFP expression in the heart. *Tg(myl7:Lifeact-GFP)^*mb21*^* transgenic embryos with no edema phenotype had a lower Lifeact-GFP fluorescent intensity in the heart (Figure 2E). In contrast, *Tg(myl7:Lifeact-GFP)^*mb22*^* transgenic embryos with a large pericardial edema showed a strong Lifeact-GFP expression (Figure 2F). *Tg(myl7:Lifeact-GFP)^*mb22*^* transgenic embryos that displayed the edema phenotype at a later stage showed moderate levels of Lifeact-GFP fluorescent intensity (Figure 2G). To confirm that the *Tg(myl7:Lifeact-GFP)^*mb22*^* transgenic line had higher levels of Lifeact-GFP expression, we compared the GFP fluorescence intensity and quantified the levels of GFP mRNA expression in cardiac muscles of three transgenic lines that showed the sever, moderate and no heart defects (Supplementary Figure 1). The data confirmed a strong correlation between the levels of Lifeact-GFP expression and the cardiac muscle defects (Supplementary Figures 1D,E). Collectively, these data indicate that high levels of Lifeact-GFP expression in cardiac myocytes could impede heart development and cardiac function, leading to pericardial edema and early lethality.

### Overexpression of Lifeact-GFP Disrupted Myofibril Organization in Cardiomyocytes of Transgenic Embryos

**FIGURE 3:**
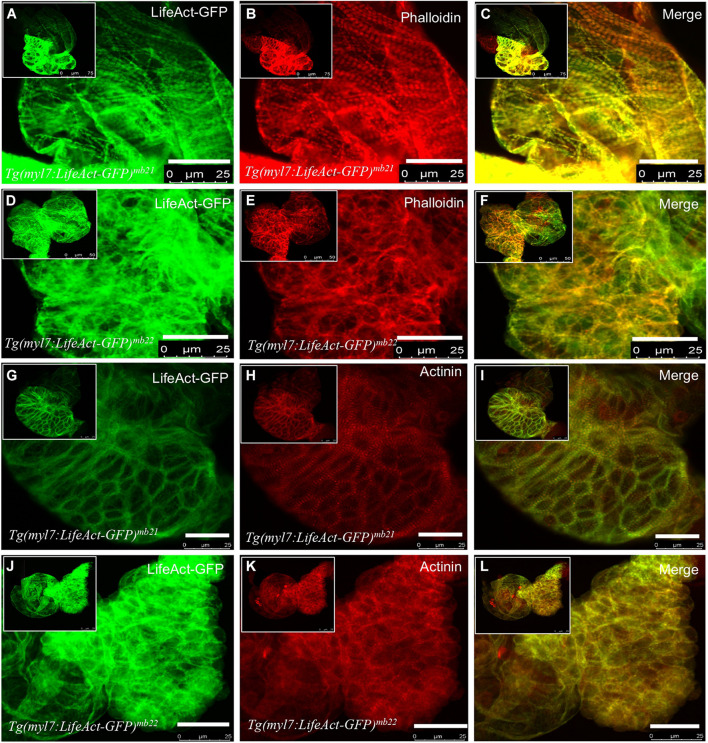
The effect of Lifeact-GFP expression on actin filament and sarcomere organization in cardiomyocytes of *Tg(myl7:Lifeact-GFP)^*mb21*^* and *Tg(myl7:Lifeact-GFP)^*mb22*^* transgenic embryos. **(A–F)** Actin think filaments revealed by Lifeact-GFP **(A,D)** and phalloidin staining **(B,E)** in *Tg(myl7:Lifeact-GFP)^*mb21*^* **(A–C)**, and *Tg(myl7:Lifeact-GFP)^*mb22*^* **(D–F)** transgenic embryos at 72 hpf. **(C,F)** are merged pictures of A-B and D-E, respectively. **(G–L)** The sarcomeric organization of actin think filament and Z-lines revealed by Lifeact-GFP **(G,J)** anti-α-actinin antibody staining **(H,K)** in *Tg(myl7:Lifeact-GFP)^*mb21*^* **(G–I)**, and *Tg(myl7:Lifeact-GFP)^*mb22*^* **(J–L)** transgenic embryos at 72 hpf. **(I,L)** are merged pictures of **(G,H,J,K)**, respectively. Scale bars: 25 μm.

To further characterize the effect of Lifeact-GFP overexpression on cardiac muscle differentiation, we compared F-actin thin filament organization in cardiomyocytes of *Tg(myl7:Lifeact-GFP)^*mb21*^* and *Tg(myl7:Lifeact-GFP)^*mb22*^* transgenic embryos by direct observation of Lifeact-GFP localization and phalloidin staining. F-actin thin filaments were clearly detected in cardiomyocytes of *Tg(myl7:Lifeact-GFP)^*mb21*^* transgenic embryos that showed no edema phenotype (Figure 3A). Staining with phalloidin revealed a co-localization with Lifeact-GFP in the F-actin thin filaments of cardiomyocytes (Figures 3B,C). In contrast, *Tg(myl7:Lifeact-GFP)^*mb22*^* transgenic embryos displayed a significant disruption of F-actin thin filaments in sarcomeres of cardiomyocytes (Figure 3D). Very few sarcomeres were detected in cardiomyocytes of *Tg(myl7:Lifeact-GFP)^*mb22*^* transgenic embryos by Lifeact-GFP (Figure 3D) or phalloidin staining (Figure 3E,F). To better quantify the myofibril defect, we compared the number of sarcomeres in a defined area of cardiac muscles in *Tg(myl7:Lifeact-GFP)^*mb21*^* and *Tg(myl7:Lifeact-GFP)^*mb22*^* transgenic embryos. The data showed that the number of sarcomeres were dramatically reduced in cardiomyocytes of the *Tg(myl7:Lifeact-GFP)^*mb22*^* transgenic larvae compared with the *Tg(myl7:Lifeact-GFP)^*mb21*^* transgenic line (Supplementary Figure 2C). Together, these data demonstrate that Lifeact-GFP overexpression in *Tg(myl7:Lifeact-GFP)^*mb22*^* disrupted F-actin filament organization in cardiomyocytes of zebrafish embryos.

F-actin thin filaments are key structures in sarcomeres of skeletal and cardiac muscles. Actin thin filaments are anchored at the Z-discs by a protein called α-actinin. To test whether disruption of actin thin filaments by Lifeact-GFP overexpression could affect the Z-line organization in the sarcomere, we next examined the Z-discs integrity using anti-α-actinin antibody staining (Figures 3G–L). The data showed that cardiomyocytes in the normal *Tg(myl7:Lifeact-GFP)^*mb21*^* transgenic line displayed striated Z-line structures (Figure 3H). However, the striated Z-lines were absent in cardiomyocytes of *Tg(myl7:Lifeact-GFP)^*mb22*^* transgenic embryos with the edema phenotype (Figure 3K). Cardiac myocytes in *Tg(myl7:Lifeact-GFP)^*mb22*^* embryos exhibited irregular F-actin structure and poor Z-line organization. Lifeact-GFP and α-actinin staining appeared as dispersed punctate within the cardiomyocytes (Figures 3J–L). Collectively, these data indicate that disruption of F-actin myofibril assembly by Lifeact-GFP overexpression could impede other sarcomere structures in cardiac myocytes.

It has been reported that overexpression of GFP could also induce cytotoxicity and apoptosis (Liu et al., 1999; Ansari et al., 2016). To test whether the defective actin filament organization could be caused by GFP overexpression, we compared the effects of GFP and Lifeact-GFP overexpression on actin filament and sarcomere organization in skeletal muscle fibers of zebrafish embryos. Zebrafish embryonic myofibers with their easy characterization provide a good model for analyzing protein cytotoxicity *in vivo*. DNA constructs expressing the Lifeact-GFP or GFP were microinjected into fertilized zebrafish embryos at 1–2 cell stages. Muscle specific expression of GFP or Lifeact-GFP was clearly detected in muscle fibers of injected embryos. Actin filament organization was characterized by phalloidin staining in the skeletal myofibers. The data showed that approximately 5% of the Lifeact-GFP expressing myofibers showed abnormal actin filament organization. In some of the injected embryos, almost half of the Lifeact-GFP expressing fibers showed actin filament defect (Supplementary Figures 3D–F). In contrast, myofibers expressing GFP appeared normal (Supplementary Figures 3A–C). These data indicate that the actin filament defect was likely caused by Lifeact rather than GFP.

### Characterization of the *Tg(myl7:Lifeact-GFP)* Integration Sites in Transgenic Lines With Heart Defect

**FIGURE 4:**
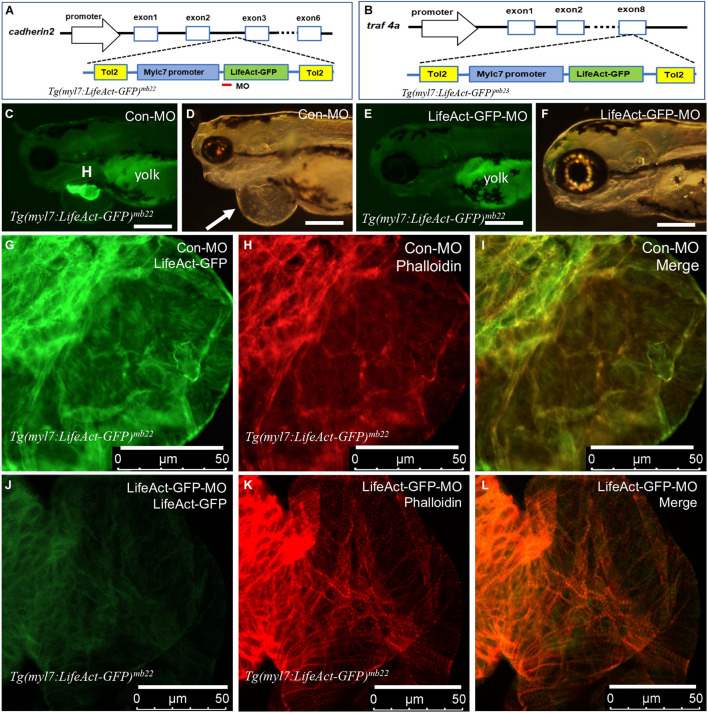
Identification of transgene integration sites in *Tg(myl7:Lifeact-GFP)^*mb22*^* and *Tg(myl7:Lifeact-GFP)^*mb23*^* transgenic embryos and the rescue of heart defect by knockdown of Lifeact-GFP expression. **(A,B)** Mapping the transgene integration sites in *Tg(myl7:Lifeact-GFP)^*mb22*^* and *Tg(myl7:Lifeact-GFP)^*mb23*^* transgenic embryos at *cadherin2* and *traf4a* genes, respectively. The Lifeact-GFP-MO is indicated by the red line. **(C–F)** Morphology of heart region of *Tg(myl7:Lifeact-GFP)^*mb22*^* transgenic embryos at 72 hpf injected with control-MO **(C,D)** and Lifeact-GFP-MO **(E,F)**, respectively. Pericardial edema is indicated by the arrow in control-MO injected *Tg(myl7:Lifeact-GFP)^*mb22*^* **(D)** transgenic mutant larvae. H, heart; Yolk shows autofluorescence. Scale bars: 250 μm. **(G–L)** Lifeact-GFP expression **(G,J)** and phalloidin staining **(H,K)** showing actin think filaments in cardiomyocytes of *Tg(myl7:Lifeact-GFP)^*mb22*^* transgenic embryos injected with control-MO **(G–I)** or Lifeact-GFP-MO **(J–L)** at 72 hpf transgenic embryos. **(I,L)** are merged pictures of **(G,H,J,K)**, respectively. Scale bars: 50 μm.

Integration of *Tg(myl7:Lifeact-GFP)* transgenes into zebrafish genome could disrupt expression and function of genes at the integration sites (Clark et al., 2004; Kawakami et al., 2004; Kawakami, 2007). To assess this potential possibility in *Tg(myl7:Lifeact-GFP)^*mb22*^* and *Tg(myl7:Lifeact-GFP)^*mb23*^* transgenic lines that displayed the edema phenotype, we mapped the transgene integration sites by inverse PCR. The data revealed that in the *Tg(myl7:Lifeact-GFP)^*mb22*^* transgenic line, the transgene was inserted into the third intron of the *cadherin2* gene (Figure 4A), which encodes a transmembrane protein named N-cadherin that has been implicated in zebrafish cardiovascular development (Bagatto et al., 2006). In the *Tg(myl7:Lifeact-GFP)^*mb23*^* transgenic line, the transgene was integrated into the last exon of *traf4a* (Figure 4B), which encodes tumor necrosis factor (TNF)-receptor–associated factor-4 (TRAF4). Previous studies showed that *Cadherin2* or *traf4a* heterozygous mutants showed normal development and could survive to adult stage without any cardiac abnormality (Brusés, 2011; Hehr et al., 2018), indicating that the cardiac muscle defects observed in *Tg(myl7:Lifeact-GFP)^*mb22*^* and *Tg(myl7:Lifeact-GFP)^*mb23*^* hemizygous transgenic lines were unlikely caused by the transgene integration in these genes.

### Knockdown of Lifeact-GFP Expression Alleviated the Heart Defect in *Tg(myl7:Lifeact-GFP)^*mb22*^* Transgenic Embryos

To confirm the heart defect in *Tg(myl7:Lifeact-GFP)^*mb22*^* transgenic zebrafish embryos was indeed caused by Lifeact-GFP expression, we decided to knock down Lifeact-GFP expression in the transgenic embryos and assess the effect on heart development. A Lifeact-GFP specific antisense morpholino (Lifeact-GFP-MO) was designed that targets the translational start site of Lifeact-GFP (Figure 4A). The MO was microinjected into fertilized eggs of *Tg(myl7:Lifeact-GFP)^*mb22*^* transgenic fish. Compared with the control MO (Con-MO) injected embryos that showed normal Lifeact-GFP expression and a strong edema phenotype (Figures 4C,D), Lifeact-GFP-MO injection dramatically reduced the Lifeact-GFP expression (Figure 4E). While the control-MO injected embryos had a pronounced edema, strikingly, knockdown of Lifeact-GFP expression completely rescued the pericardiac edema phenotype in the Lifeact-GFP-MO injected transgenic embryos (Figure 4F), suggesting that edema phenotype was indeed caused by Lifeact-GFP overexpression.

To better characterize the heart phenotype and rescue, we analyzed F-actin thin filaments in cardiomyocytes of MO injected embryos by Lifeact-GFP localization and phalloidin staining. As shown in Figure 4G, a heart from control-MO injected embryos showed strong Lifeact-GFP expression. However, no F-actin myofibrils could be detected in cardiomyocytes of the control-MO injected embryos (Figures 4H,I). In contrast, in Lifeact-GFP-MO injected embryos, Lifeact-GFP expression was dramatically reduced (Figure 4J). Phalloidin staining revealed highly organized thin filaments in cardiomyocytes of the Lifeact-GFP knockdown embryos (Figures 4K,L). To quantify the myofibril recovery, we compared the number of sarcomeres in cardiomyocytes of *Tg(myl7:Lifeact-GFP)^*mb22*^* transgenic embryos injected with Con-MO and Lifeact-GFP-MO. Compared with the Con-MO injected embryos, the number of sarcomeres was dramatically increased in cardiomyocytes of Lifeact-GFP-MO injected embryos (Supplementary Figure 4). Collectively, these data indicate that Lifeact-GFP overexpression could disrupt F-actin filament organization in cardiomyocytes of zebrafish embryos, and thus the use of Lifeact-GFP requires caution and evaluation of the transgenic model to avoid artifacts.

## Discussion

In this study, we generated *Tg(myl7:Lifeact-GFP)* transgenic zebrafish models to study cardiomyopathy in *smyd1b* mutants and to investigate the effect of Lifeact-GFP overexpression on heart development. We showed that Lifeact-GFP transgenic zebrafish is a useful model for studying contractile dynamics of F-actin filaments in cardiomyocytes *in vivo*. Using this model, we found that loss of *smyd1b* resulted in poor actin filament organization in cardiac myocytes. Moreover, our studies also demonstrated that strong Lifeact-GFP expression was detrimental to F-actin filament organization in cardiomyocytes, causing pericardial edema and early embryonic lethality of the transgenic embryos. Collectively, these data indicate that although Lifeact-GFP is a powerful probe for studying actin dynamic in cardiomyocytes *in vivo*, Lifeact-GFP could alter actin filament arrangement and dynamics in cardiomyocytes. Lifeact-GFP mediated artifacts are concentration-dependent and thus the use of Lifeact requires a closer examination and optimization to minimize the Lifeact-GFP induced adverse effects.

### Cardiomyopathy in *smyd1b* Mutants

Using Lifeact-GFP, we found that *smyd1b* is required for actin filament assembly in cardiac myocytes of zebrafish embryos. This finding is consistent with data from previous studies using phalloidin and immunostaining that showed that Smyd1 is required for sarcomere assembly in skeletal and cardiac muscles (Tan et al., 2006; Just et al., 2011; Li et al., 2013; Prill et al., 2015; Jiao et al., 2021). The molecular mechanism of Smyd1 function in sarcomere assembly is not well known. Actin thin filament is a key component of the sarcomere. Thin filaments work together with thick filaments for generation and propagation of mechanical force. It has been reported that Smyd1 is a myosin heavy chain binding protein (Just et al., 2011). Biochemical analysis revealed that myosin heavy chain (MHC) and several other muscle proteins are methylated at lysine residues (Huszar and Elzinga, 1969; Hardy et al., 1970; Huszar, 1972; Tong and Elzinga, 1983; Iwabata et al., 2005). Given that Smyd1b is a lysine methyltransferase, Smyd1 may directly methylate MHC and possibly other muscle proteins critical to protein folding and stability. However, the methylation targets of Smyd1 have yet to be identified. Our previous studies have demonstrated that Smyd1 is required for MHC protein expression and stability in skeletal and cardiac muscle cells (Li et al., 2013; Jiao et al., 2021), and that loss of myosin heavy chain abolished sarcomere assembly, including actin thin filaments in zebrafish embryonic skeletal muscles (Xu et al., 2012; Li et al., 2020). We cannot rule out the possibility that the defective F-actin assembly in cardiomyocytes of *smyd1b* mutant mutants was a secondary defect from loss of MHC in *smyd1b* mutants.

Smyd1 function in cardiac muscles is conserved during evolution from fish to human. Recent studies demonstrated that genetic mutations in human *SMYD1* are associated with dilated cardiomyopathy and hypertrophic cardiomyopathy (Coyan et al., 2019; Fan et al., 2019). An infant patient carrying a *SMYD1* mutation suffered from dilated cardiomyopathy and needed heart transplantation at the infant stage (Coyan et al., 2019). A peripheral muscle biopsy showed disorganized muscle fibers with minimal mitochondria (Coyan et al., 2019). The sarcomere and mitochondria defects resemble the heart defects from zebrafish and mouse Smyd1 mutant models (Tan et al., 2006; Just et al., 2011; Rasmussen et al., 2015; Warren et al., 2018). In mice, knockout of the *Smyd1* gene results in death of the fetus or embryos and various failures in the development of the cardiac and vascular system (Gottlieb et al., 2002; Rasmussen et al., 2015; Nagandla et al., 2016; Stewart et al., 2016). In zebrafish, loss of *smyd1* causes edema and no muscle contraction (Just et al., 2011; Cai et al., 2019; Jiao et al., 2021). *Tg(myl7:Lifeact-GFP)* transgenic zebrafish could be a useful model for future studies of cardiomyopathy from Smyd1 deficiency.

### Disruption of α-Actin Filament Organization by Lifeact-GFP

Data from this study showed that overexpression of Lifeact-GFP disrupted actin filament organization in cardiac myocytes, leading to pericardial edema and early larval lethality. This adverse effect was not observed in a previous study using the same *Tg(myl7:Lifeact-GFP)* transgene (Reischauer et al., 2014). Reischauer and colleagues showed that expression of Lifeact-GFP in zebrafish heart did not interfere with cardiac muscle development. We speculate that the difference might be due to varied levels of Lifeact-GFP expression in different transgenic lines. Indeed, we observed in the same study here that six of the eight *Tg(myl7:Lifeact-GFP)* transgenic founders could produce healthy and viable transgenic offspring. All these six transgenic lines had lower levels of Lifeact-GFP expression. The other two lines with higher levels of Lifeact-GFP expression developed edema and were early embryonic lethal. The varied levels of gene expression were likely due to the positional effect of transgene integration. We have mapped the integration sites in *Tg(myl7:Lifeact-GFP)^*mb22*^* and *Tg(myl7:Lifeact-GFP)^*mb23*^* to the *cadherin2* and *traf4a* genes, respectively. We do not think the heart phenotype in these lines was caused by disruption of these host genes at the integration sites because the heterozygous mutant of *cadherin2* or *traf4a* in zebrafish showed no edema phenotypes (Brusés, 2011; Hehr et al., 2018), and moreover, the edema phenotype in *Tg(myl7:Lifeact-GFP)^*mb22*^* could be rescued by knockdown of Lifeact-GFP expression.

It has been reported that higher levels of Lifeact-GFP expression could influence the assembly of actin skeleton in cell culture, yeast, *Drosophila*, and plant cells (Van Der Honing et al., 2011; Spracklen et al., 2014; Courtemanche et al., 2016). Strong expression of Lifeact-GFP in *Drosophila* disrupted F-actin remodeling, and resulted in female fertility defects (Spracklen et al., 2014). In fission yeast, it was shown that Lifeact altered actin assembly during endocytosis and cytokinesis (Courtemanche et al., 2016). Recent studies showed that Lifeact also induced dose-response effects on F-actin assembly in human Mesenchymal Stem Cells (Flores et al., 2019), and transient transfection of Lifeact-GFP was toxic to C2C12 myoblast cells (Yu, 2013). Most of these previous studies were primarily focused on Lifeact-GFP induced artifacts in cytoskeleton structures of non-muscle cells. Data from this study demonstrate that Lifeact can induce defective actin myofibril organization in the sarcomeres of skeletal and cardiac muscle cells.

The molecular structure of Lifeact and its basis for inducing actin filament artifacts have been recently investigated (Belyy et al., 2020; Kumari et al., 2020). Structural analysis of the Lifeact–F-actin complex showed that Lifeact interacts with a hydrophobic binding pocket on F-actin (Belyy et al., 2020; Kumari et al., 2020). This hydrophobic binding pocket is required for myosin binding. Lifeact could compete with myosin for binding with actin, thus providing a mechanistic explanation for the adverse effects of Lifeact on cell morphology and muscle cell differentiation *in vivo*. However, previous studies have shown that excessive Lifeact stabilizes actin filaments and bundles even in the absence of Myosins (Courtemanche et al., 2016). Structural analysis revealed that Lifeact interacts with a hydrophobic binding pocket on F-actin and stretches over 2 adjacent actin subunits, stabilizing the DNase I-binding loop (D-loop) of actin in the closed conformation (Belyy et al., 2020; Kumari et al., 2020). D-loop plasticity has been implicated in filament formation and stability (Durer et al., 2012; Grintsevich et al., 2017; Das et al., 2020). Thus, an alternative possibility of Lifeact action on actin filament disruption could be contributed by locking D-loop in the closed conformation.

In addition to Lifeact, several other actin probes have been developed using actin binding domains from other proteins or actin nanobody for live imaging. It has been reported that in contrast to Lifeact, F-tractin does not alter actin rearrangement during Drosophila follicle development (Spracklen et al., 2014). However, F-tractin expression could perturb actin cytoskeleton organization in a Xenopus cell line (Belin et al., 2014). Compared with Lifeact, F-tractin is larger in size, it might interfere with actin binding with other regulatory or accessory proteins. Actin-Chromobody represents another novel approach to visualize actin dynamic assembly in living cells. Chromobodies combine the specificity of antibodies with the convenience of live fluorescence imaging, which can be easily expressed by a DNA construct (Panza et al., 2015). It appears that high levels of actin-Chromobody expression does not alter actin dynamics (Schiavon et al., 2020). Chromobodies have been successfully applied to visualize actin in mammalian nuclei (Plessner et al., 2015), in tobacco leaf cells (Rocchetti et al., 2014) and zebrafish (Panza et al., 2015). These alternative actin probes could be evaluated in zebrafish embryonic cardiac and skeletal muscles to determine their cytotoxicity *in vivo*.

In summary, our studies show that *Tg(myl7:Lifeact-GFP)* transgenic zebrafish is a useful model to study contractile dynamics of F-actin filaments in cardiomyocytes *in vivo* and to investigate cardiomyopathy from defective thin filament organization. Data from this study also demonstrated that strong Lifeact-GFP expression in cardiomyocytes could alter actin filament arrangement and dynamics in cardiomyocytes causing heart development defect and embryonic lethality. The Lifeact-GFP induced artifacts are concentration-dependent and thus the use of Lifeact requires careful evaluation of the transgenic model to minimize the adverse effects.

## Data Availability Statement

The raw data supporting the conclusions of this article will be made available by the authors, without undue reservation.

## Ethics Statement

The animal study was reviewed and approved by the Institutional Animal Care and Use Committee of University of Maryland Baltimore.

## Author Contributions

RX and SD conceived and designed the research project and analyzed the data. RX performed the experiments, data collection, and wrote the first draft of the manuscript. SD revised the manuscript. Both authors read and approved the final manuscript.

## Conflict of Interest

The authors declare that the research was conducted in the absence of any commercial or financial relationships that could be construed as a potential conflict of interest.

## Publisher’s Note

All claims expressed in this article are solely those of the authors and do not necessarily represent those of their affiliated organizations, or those of the publisher, the editors and the reviewers. Any product that may be evaluated in this article, or claim that may be made by its manufacturer, is not guaranteed or endorsed by the publisher.
