# Supplementary material for: Overexpression of Lifeact-GFP Disrupts F-Actin Organization in Cardiomyocytes and Impairs Cardiac Function
Source: Front Cell Dev Biol. 2021 Oct 26;9:746818. doi: 10.3389/fcell.2021.746818 (PMC8576398; doi:10.3389/fcell.2021.746818)
Supplement: Supplementary file 1 [file Data_Sheet_1.pdf]

Supplementary Material

Overexpression of LifeActLifeact-GFP disrupts F-actin organization in cardiomyocytes and impairs cardiac function

Rui Xu, and Shaojun Du*
Department of Biochemistry and Molecular Biology, Institute of Marine and Environmental Technology, University of Maryland School of Medicine, Baltimore, MD 21202, USA

Fig. S1. Quantification of GFP expression in transgenic embryos
A-C. GFP expression in heart tube of *Tg(myl7:Lifeact-GFP)*^{mb22} (A), *Tg(myl7:Lifeact-GFP)*^{mb23} (B), and *Tg(myl7:Lifeact-GFP)*^{mb21} (C) transgenic embryos at 24 hpf, respectively.
D and E. Comparison of GFP fluorescence intensity and GFP mRNA expression in *Tg(myl7:Lifeact-GFP)*^{mb22}, *Tg(myl7:Lifeact-GFP)*^{mb23}, and *Tg(myl7:Lifeact-GFP)*^{mb21} transgenic embryos at 28 hpf. Scale bars: 300 μm.

Fig. S2. The effect of Lifeact-GFP expression on sarcomere formation in cardiomyocytes of transgenic embryos
A and B. Phalloidin staining shows sarcomere thin filaments in *Tg(myl7:Lifeact-GFP)*^{mb21} (A), and *Tg(myl7:Lifeact-GFP)*^{mb22} (B) transgenic embryos at 72 hpf. The white boxes represent the defined areas used in sarcomere calculation. Scale bars: 50 μm.
C. Comparison of the number of sarcomeres in cardiomyocytes of *Tg(myl7:Lifeact-GFP)*^{mb21} (A), and *Tg(myl7:Lifeact-GFP)*^{mb22} (B) transgenic embryos.

Fig. S3. Comparison of Lifeact-GFP and GFP expression in skeletal myofibers of zebrafish embryos
DNA constructs expressing Lifeact-GFP or GFP were microinjected into fertilized zebrafish embryos. Muscle specific GFP (A-C) or Lifeact-GFP (D-F) expression and actin filament organization were characterized by phalloidin staining in the skeletal myofibers. Defective actin filament organization was clearly detected in some myofibers (indicated by *) of the Lifeact-GFP injected embryo (D-F). Scale bars: 25 μm.

Fig. S4. The effect of Lifeact-GFP expression on sarcomere formation in cardiomyocytes of transgenic embryos
A and B. Phalloidin staining shows sarcomere thin filaments in *Tg(myl7:Lifeact-GFP)*^{mb22} transgenic embryos injected with Control-MO (A) or Lifeact-GFP-MO (B) at 72 hpf. The white boxes represent the defined areas used in sarcomere calculation. Scale bars: 50 μm.
C. Comparison of the number of sarcomeres in cardiomyocytes of Control-MO (Control) and Lifeact-GFP-MO (Knockdown) injected *Tg(myl7:Lifeact-GFP)*^{mb22} transgenic embryos.

Fig. S1

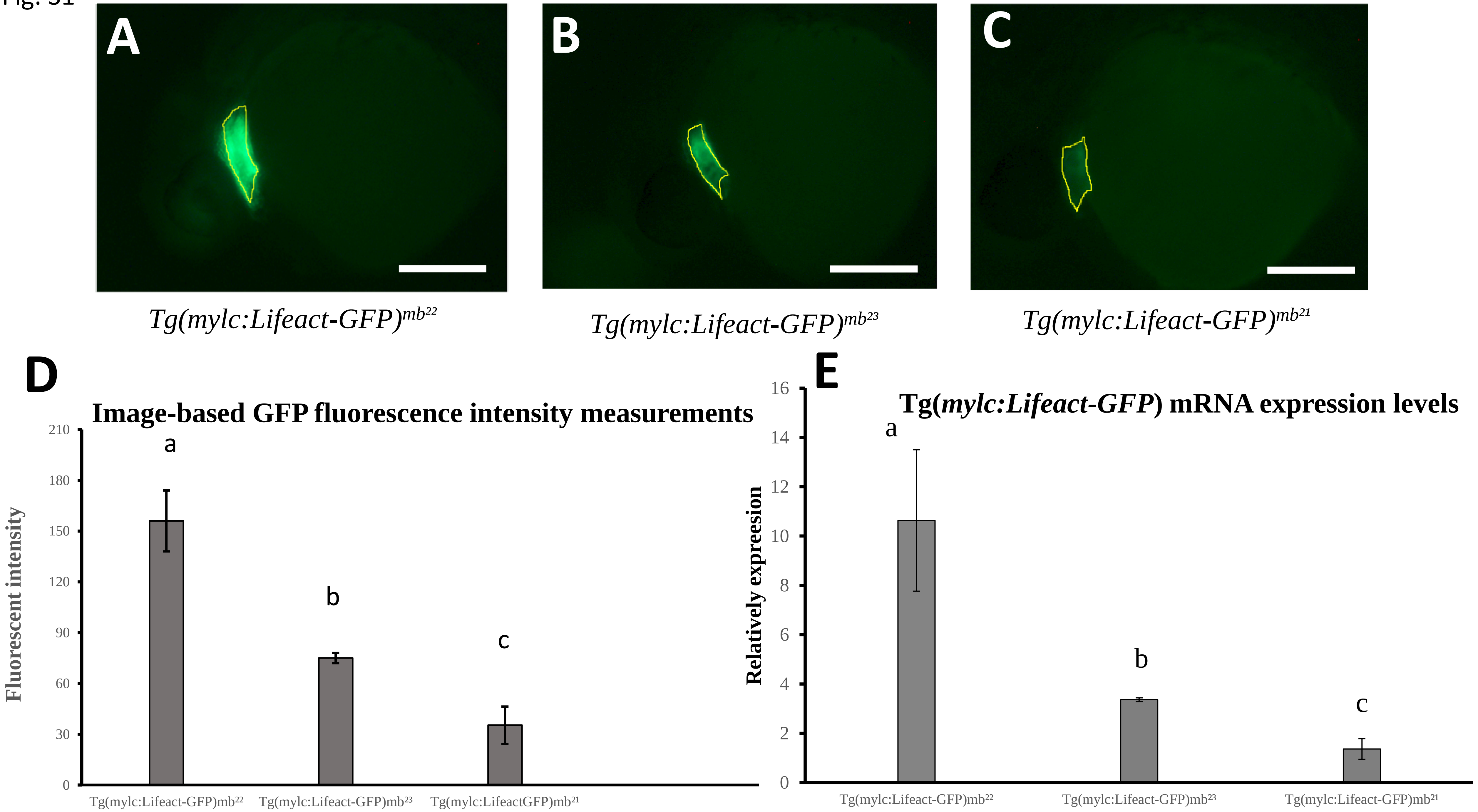

A
B
C
Tg(mylc:Lifeact-GFP)mb22
Tg(mylc:Lifeact-GFP)mb23
Tg(mylc:Lifeact-GFP)mb21
D
Image-based GFP fluorescence intensity measurements
Fluorescent intensity
210
180
150
120
90
60
30
0
a
b
c
Tg(mylc:Lifeact-GFP)mb22
Tg(mylc:Lifeact-GFP)mb23
Tg(mylc:LifeactGFP)mb21
E
Tg(mylc:Lifeact-GFP) mRNA expression levels
Relatively expreesion
16
14
12
10
8
6
4
2
0
a
b
c
Tg(mylc:Lifeact-GFP)mb22
Tg(mylc:Lifeact-GFP)mb23
Tg(mylc:Lifeact-GFP)mb21

Fig. S2

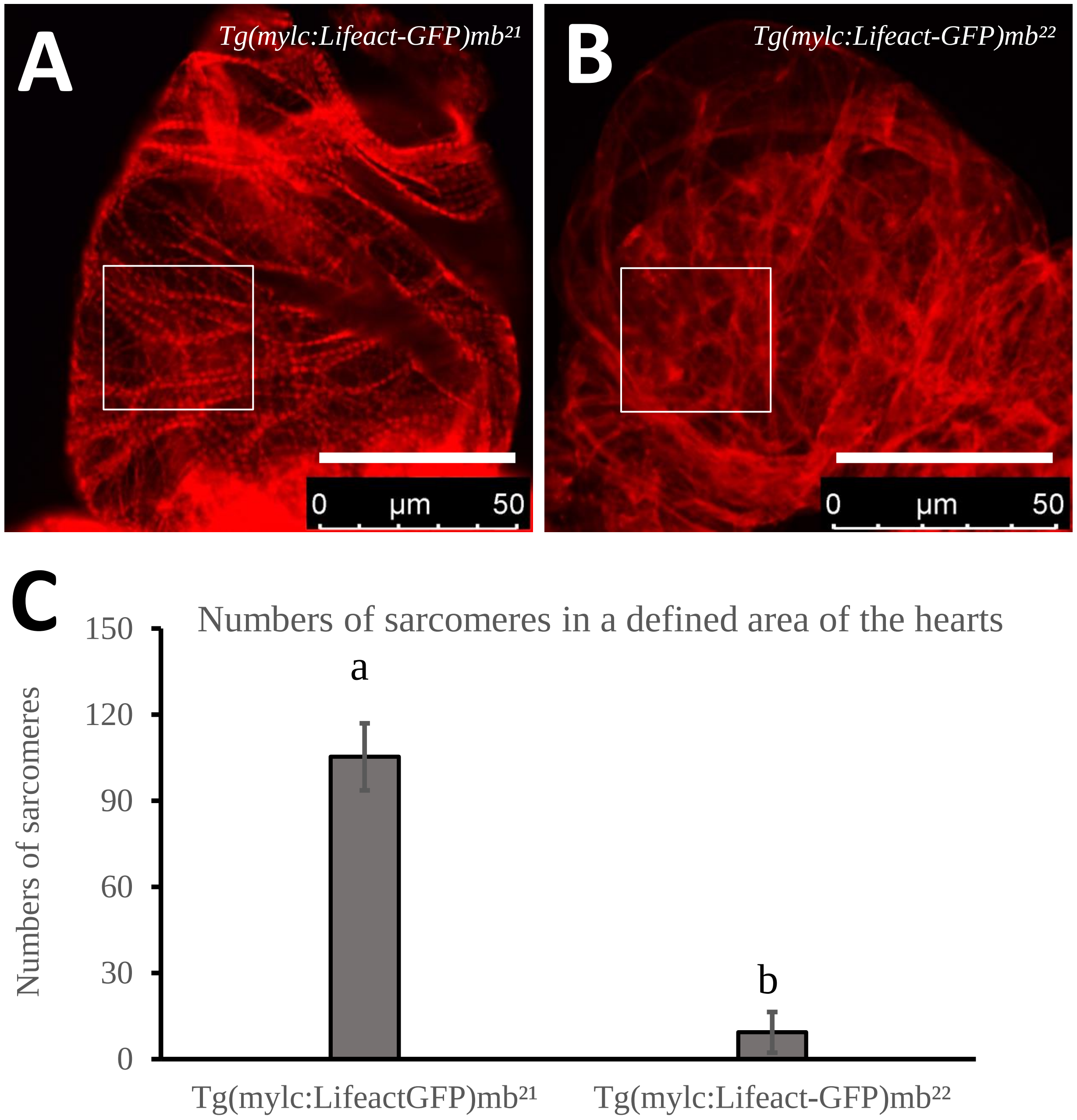

A
Tg(mylc:Lifeact-GFP)mb21
0 µm 50
B
Tg(mylc:Lifeact-GFP)mb22
0 µm 50
C
Numbers of sarcomeres in a defined area of the hearts
Numbers of sarcomeres
150
120
90
60
30
0
a
b
Tg(mylc:LifeactGFP)mb21
Tg(mylc:Lifeact-GFP)mb22

Fig. S3

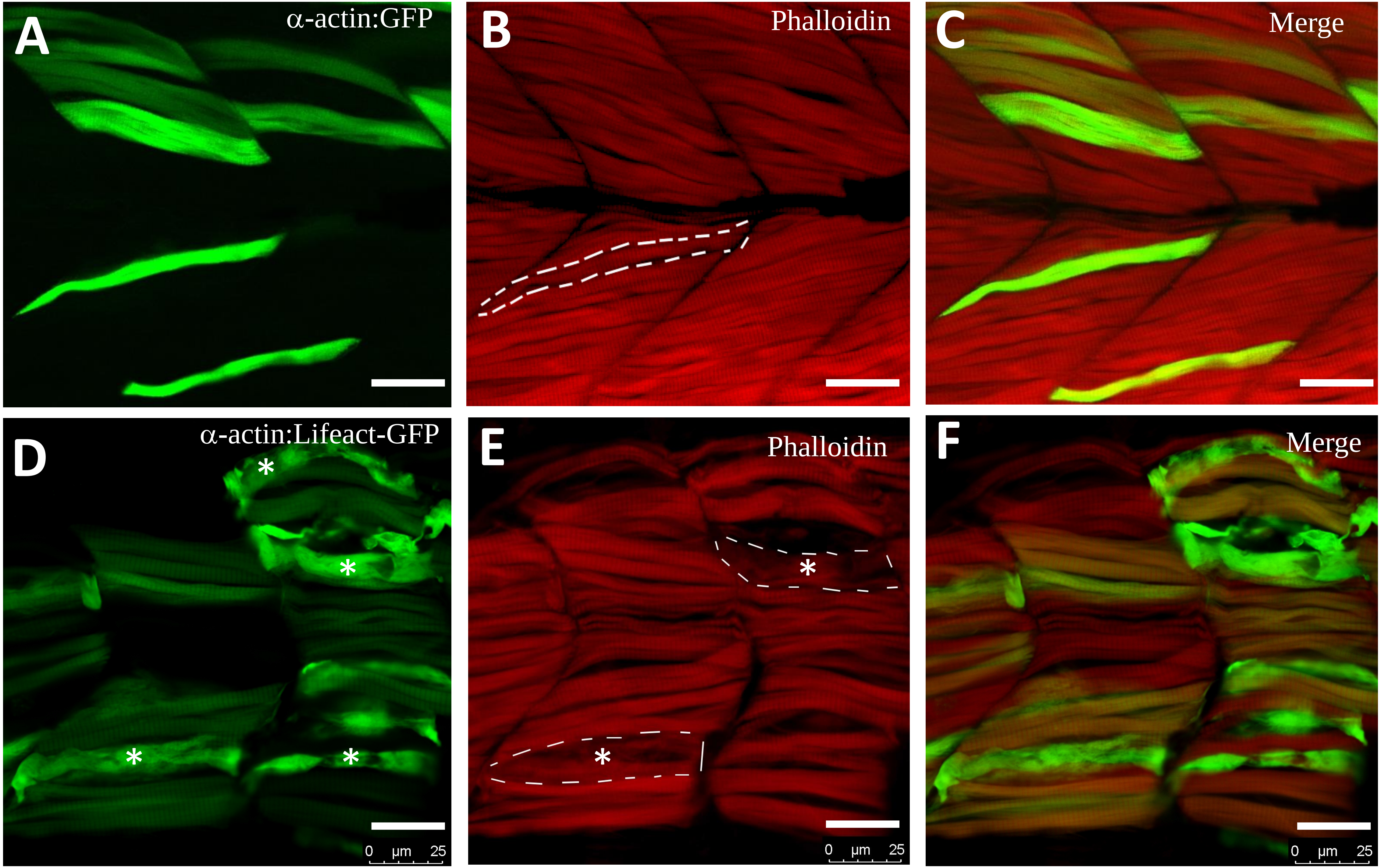
A
α-actin:GFP
B
Phalloidin
C
Merge
D
α-actin:Lifeact-GFP
*
*
*
*
0 μm 25
E
Phalloidin
*
*
0 μm 25
F
Merge
0 μm 25

Fig. S4

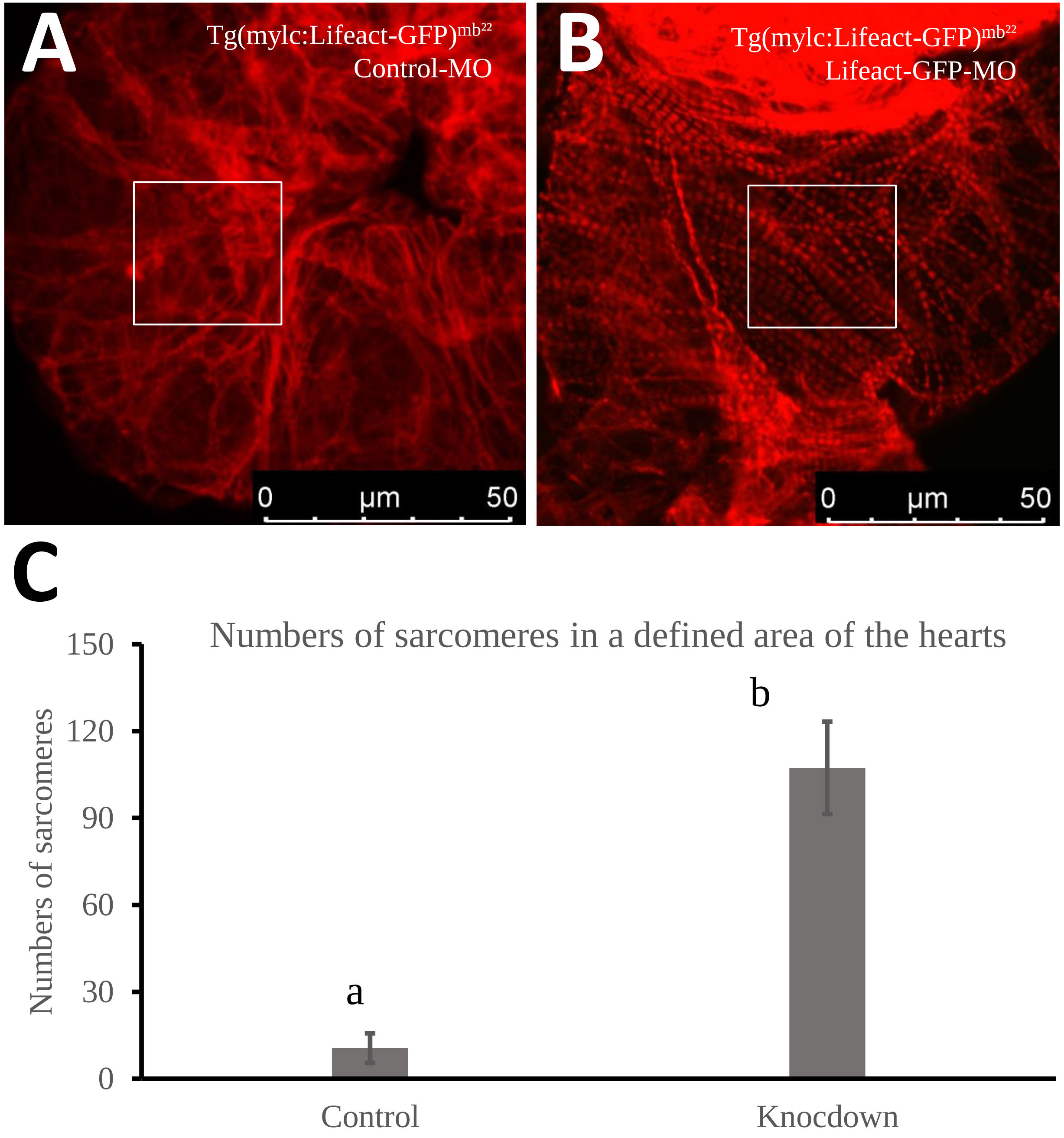

A
Tg(mylc:Lifeact-GFP)mb22
Control-MO
0 μm 50
B
Tg(mylc:Lifeact-GFP)mb22
Lifeact-GFP-MO
0 μm 50
C
Numbers of sarcomeres in a defined area of the hearts
Numbers of sarcomeres
150
120
90
60
30
0
a
b
Control
Knocdown
